# Development and Validation of an Ammonia-Resistant Thermal-Conductivity Analyzer for In Situ Monitoring of NH_3_-Cracking-Processes

**DOI:** 10.3390/s26175463

**Published:** 2026-08-28

**Authors:** Lucas Ott, Ottfried Schmidt, Hans-Peter Rabl

**Affiliations:** Technology Campus (TC) Kelheim, Ostbayerische Technische Hochschule (OTH) Regensburg, Hopfenbachweg 4, 93309 Kelheim, Germanyhans-peter.rabl@oth-regensburg.de (H.-P.R.)

**Keywords:** ammonia cracking, thermal conductivity detection, gas analysis, hydrogen carrier, hydrogen monitoring, sensor validation

## Abstract

Ammonia is increasingly considered a promising hydrogen carrier due to its high hydrogen density and well-established infrastructure. Monitoring ammonia cracking efficiency requires robust, continuous gas analysis across a wide concentration range, with resistance to corrosive gases. Conventional methods such as gas chromatography or mass spectrometry meet these requirements, but are costly and operationally complex. To address this gap, a Thermal Conductivity Analyzer (TCA) was developed based on an OEM module and validated for continuous in situ monitoring of ammonia cracking. The system includes a pump-driven bypass extraction line and a three-stage calibration procedure: zero-point correction, look-up table generation, and span calibration. Measurement stability was assessed using binary H_2_/N_2_ mixtures and a quasi-binary surrogate of the ammonia cracking product gas, mixed via mass flow controllers (MFCs). The analyzer was then applied to characterize a monolithic ammonia cracking catalyst from 200–650 °C. With daily zero-point and span calibration, all measured H_2_ concentrations fell within the mixing uncertainty of the MFCs across 0–100 vol.%. For the synthetic cracking gas, maximum conversion ratio deviations of +0.317 and −0.224 percentage points were achieved. These results demonstrate that the TCA offers a simple, ammonia-resistant alternative for monitoring NH_3_ cracking processes, with uncertainties competitive with MFC repeatability.

## 1. Introduction

In order to combat the ongoing challenge posed by climate change, fossil fuels must be avoided wherever possible or replaced by renewable, CO_2_-free alternatives where feasible. Hydrogen plays a particularly important role in this context. Hydrogen carriers are used to transport hydrogen over long distances, offering several advantages over molecular hydrogen [1]. Over the last decades ammonia has emerged as a promising candidate for use as a hydrogen transport molecule [2,3]. The key arguments in favor of ammonia as a hydrogen carrier are its high hydrogen density (17.7 wt.% H_2_) [4,5,6], its comparatively easy liquefiability [7], its well-established production [8,9] and distribution infrastructure [10] and the possibility of carbon-free production [11]. In order to extract hydrogen from the ammonia, it must be decomposed into hydrogen and nitrogen. This process can be carried out autothermally [12,13], thermally [13,14,15], or by means of plasma-based methods [16,17]. All of these methods require precise concentration measurement in the reactor product gas in order to assess the efficiency of the cracking process. This measurement poses a number of challenges: the analyzer must cover a wide concentration range and withstand the aggressive and corrosive nature of ammonia and hydrogen. Currently, product gas composition is typically determined using mass spectrometry [18] or gas chromatography, [19] often in combination with titration [20] or infrared spectroscopy [21]. More experimental approaches also exist: Ruzaikin and Lukashov [22] employ a thermohydraulic method to measure cracker product gas concentrations, while Andersen et al. [23] measure the product volumetric flow and apply a volumetric balance to estimate the conversion ratio. In this paper, thermal conductivity measurement is introduced as a robust and complementary method for monitoring the efficiency of ammonia decomposition. Owing to the pronounced contrast in thermal conductivity between hydrogen and both nitrogen and ammonia [24], the technique enables concentration measurements across a wide dynamic range while remaining inherently insensitive to chemically aggressive gas components, as the gas contacts only highly resistant materials such as PTFE, platinum, sapphire glass, and stainless steel. As a non-consumptive, continuously operating method, thermal conductivity measurement offers a simple and durable alternative to established analytical techniques for monitoring ammonia cracking processes. While thermal conductivity detection is an established method for related applications, no quantitative performance data validated under ammonia-cracking conditions could be identified in the literature. This work addresses this gap by presenting a thermal-conductivity-based gas analyzer validated against synthetic cracking gas compositions.

## 2. Materials and Methods

**Figure 1:**
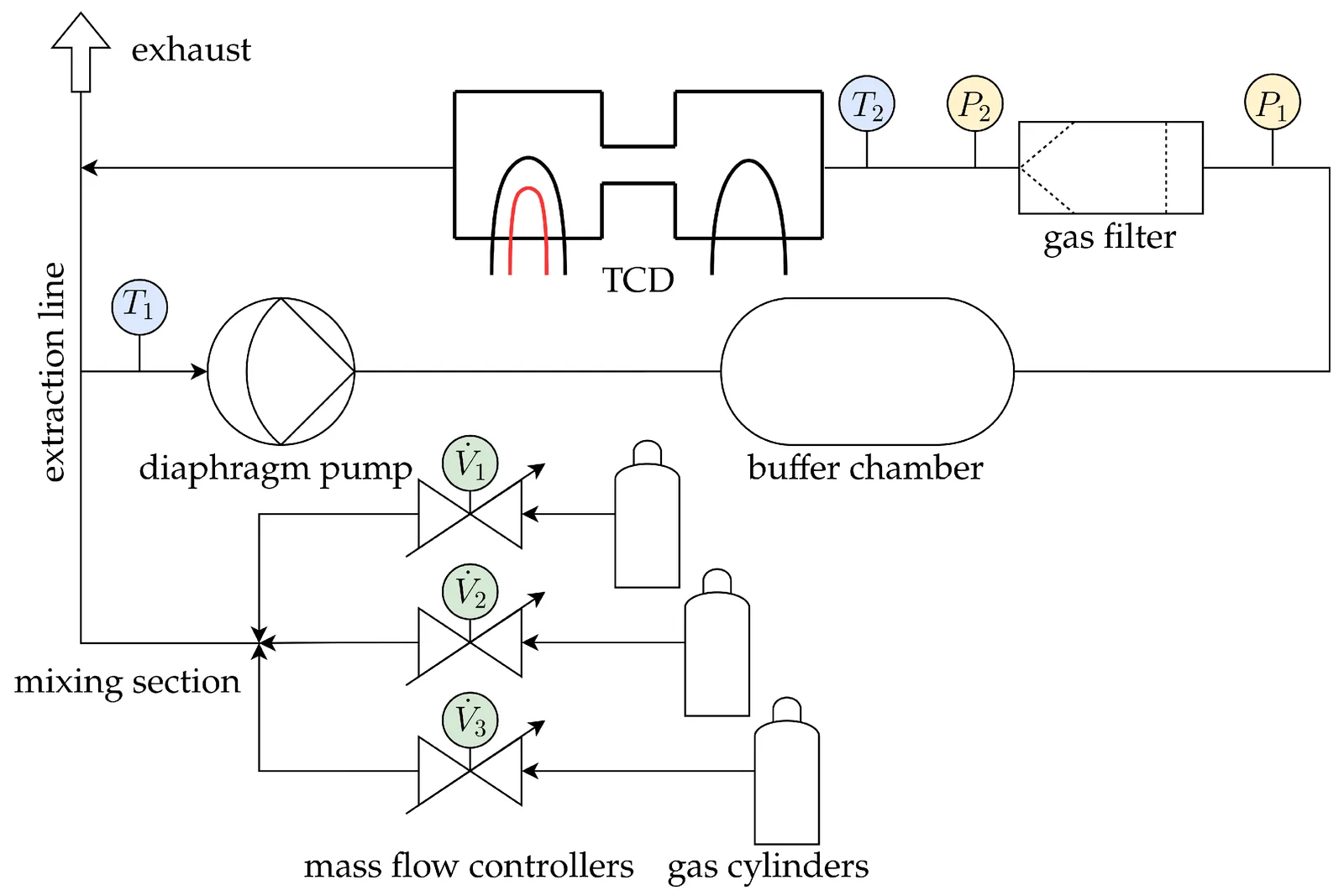
The complete gas path of the experimental setup. The gas is premixed by MFCs and subsequently directed into the extraction system, where a portion is extracted and preconditioned for thermal conductivity measurement.

The layout of the experimental setup is shown in Figure 1. Mass flow controllers (MFCs) (Bronkhorst ElFlow Prestige FG-201CV for NH_3_ and Bronkhorst ElFlow Prestige FG-211CV for all other gases, Bronkhorst High-Tech B.V., Ruurlo, The Netherlands) are used to meter and mix gases at specified volume flow rates. After a mixing section the gas is fed directly into an extraction line. A fixed volume flow of $1.0\;\frac{l}{\min}$ is then drawn from this line using a diaphragm pump (KNF NMP820KPDC, KNF Micro AG, Reiden, Switzerland) and preconditioned for thermal conductivity measurement. This bypass arrangement is chosen to account for the variation in total flow rate that occurs in an actual cracking application, as the flow rate depends on the conversion ratio. Before reaching the pump, the gas passes a PT1000A resistance thermometer (003-KS-PT1000A-4L-1.0-330, otom Group GmbH, Braeunlingen, Germany), that monitors the gas temperature. If gas temperature exceeds 50 °C, the control system shuts off the pump to protect it from thermal damage. Downstream of the pump, the gas enters a buffer chamber with a volume of 83 mL, which attenuates the pressure oscillations caused by the pump. The gas is then passed through a filter with a pore size of 2 μm to ensure it is free of particles. The filter is pressure-monitored on both sides with piezoresistive sensors (EPT 7100, Variohm Eurosensor, Heidelberg, Germany) allowing both the absolute system pressure and any filter blockage to be determined from the absolute reading and differential pressure respectively. The Thermal Conductivity Detector (TCD) (OEM-TCD, LFE GmbH, Bruchkoebel, Germany) is then reached. All wetted components of the gas path are constructed from ammonia-resistant materials (PPS, EPDM, PTFE, stainless steel 316 and 321, Al_2_O_3_, sapphire, glass, and SiO*_x_*-coated platinum filaments), thereby ensuring the chemical compatibility and long-term operational stability of the measurement system under NH_3_-containing process conditions.

**Figure 2:**
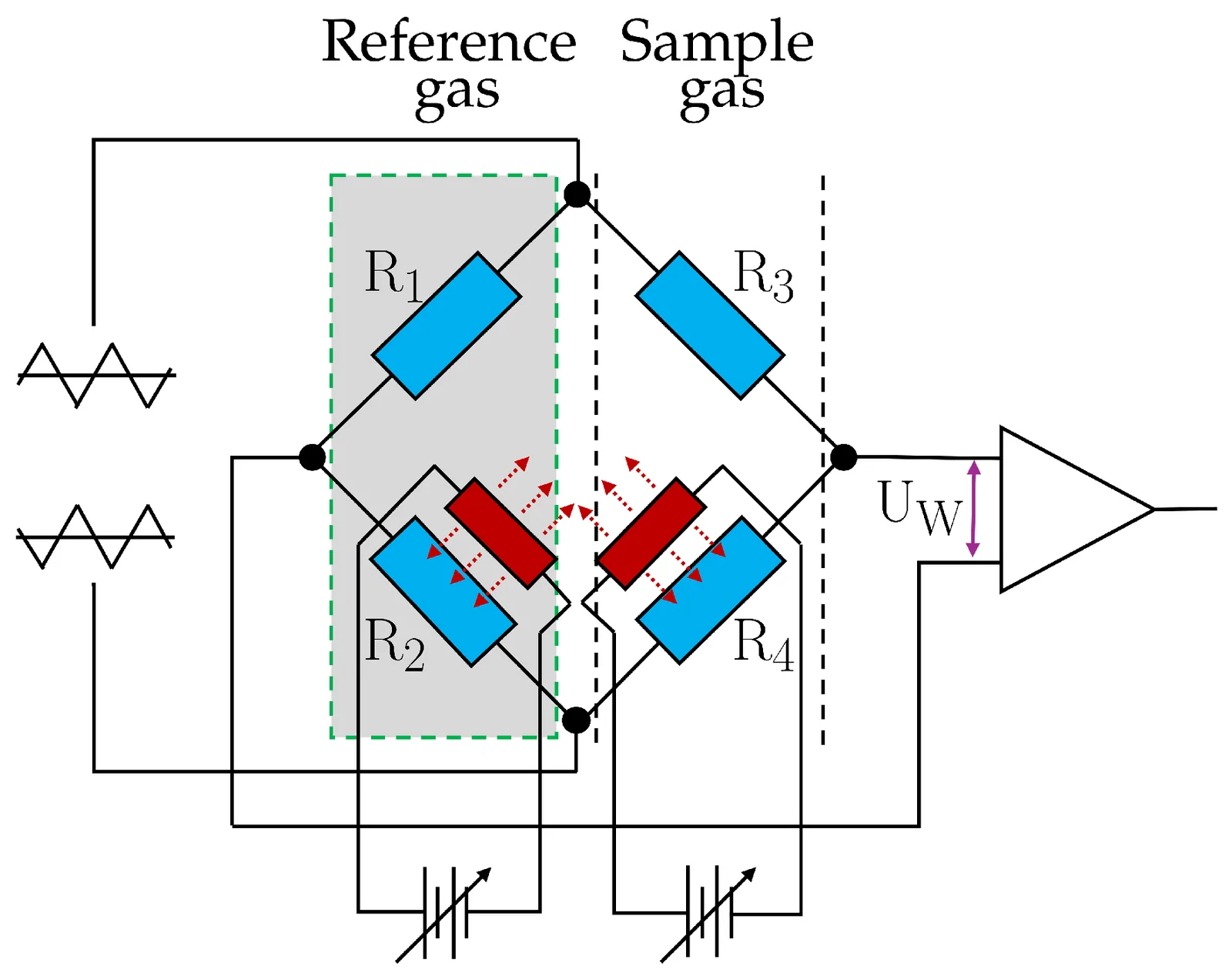
Layout of the measurement cells within the LFE OEM TCD. Both the reference gas path and the sample gas path each contain one active and one passive cell. The reference cells are filled with N_2_ and hermetically sealed by the manufacturer. The heating currents of the two active cells can be adjusted independently, allowing the bridge voltage to be zeroed physically and the measurement sensitivity to be adjusted. (Figure adapted from [25]).

The detector uses a Wheatstone bridge to compare the thermal conductivity of the process gas, measured by an active/passive cell pair, to that of pure nitrogen, measured by an analogous reference cell pair, and outputs a raw signal correlated with the gas composition of the process gas. This comparison is achieved by measuring the temperatures within these cells. The wall of each cell is maintained at a constant temperature. The passive cells contain a sensing filament that measures the gas temperature, while the active cells contain both a heating filament and a sensing filament, with the sensing filament positioned between the heating filament and the cell wall. The heating currents in the measurement and reference active cells can be adjusted independently to zero the bridge and set the measurement sensitivity. The wiring of the cells within the bridge circuit is shown in Figure 2. The TCD is recalibrated at this base level only when the thermal conductivity of the gas to be measured differs significantly from that of previously measured gases, in which case the resulting calibration values are stored for reuse.

Prior to each measurement campaign, the TCA is calibrated by applying the gas (mixture) with the lowest thermal conductivity among all gases present in the process as the calibration reference. This gas is referred to as the zero gas. While the zero gas is being applied, the detector’s raw value is read and stored as the zero-point offset (ZPO) value. In all subsequent measurements, this ZPO is subtracted from every raw value to yield the zeroed value. The zeroed value serves as a basis for constructing a look-up table (LUT). To this end, various mixtures of the target gas at different concentrations are generated using the MFCs, and their corresponding zeroed values are mapped to the desired output quantity. Via the LUT, the zeroed value can thus be related not only to gas concentrations but to any gas parameters to which a unique raw value can be unambiguously assigned. In all experiments described below, 11 equidistant breakpoints are used, between which linear interpolation is performed. Sensitivity drift is compensated by applying a span calibration, in which a gain factor is multiplied by the zeroed value. The gain factor is determined by passing a gas of known output value, distinct from that of the zero gas, through the TCA. This gas is referred to as the span gas. The gain factor corresponds to the ratio of the expected zeroed value of the span gas, obtained from the inverse look-up table (ILUT) of the desired output value, to the actually measured zeroed value, according to the following relationship:

(1) $$\mathrm{gain}=\frac{\mathrm{ILUT}(\mathrm{desired}\;{\mathrm{output}}_{\mathrm{span}\;\mathrm{gas}})}{{\mathrm{zeroed}}_{\mathrm{span}\;\mathrm{gas}}}.$$

**Figure 3:**
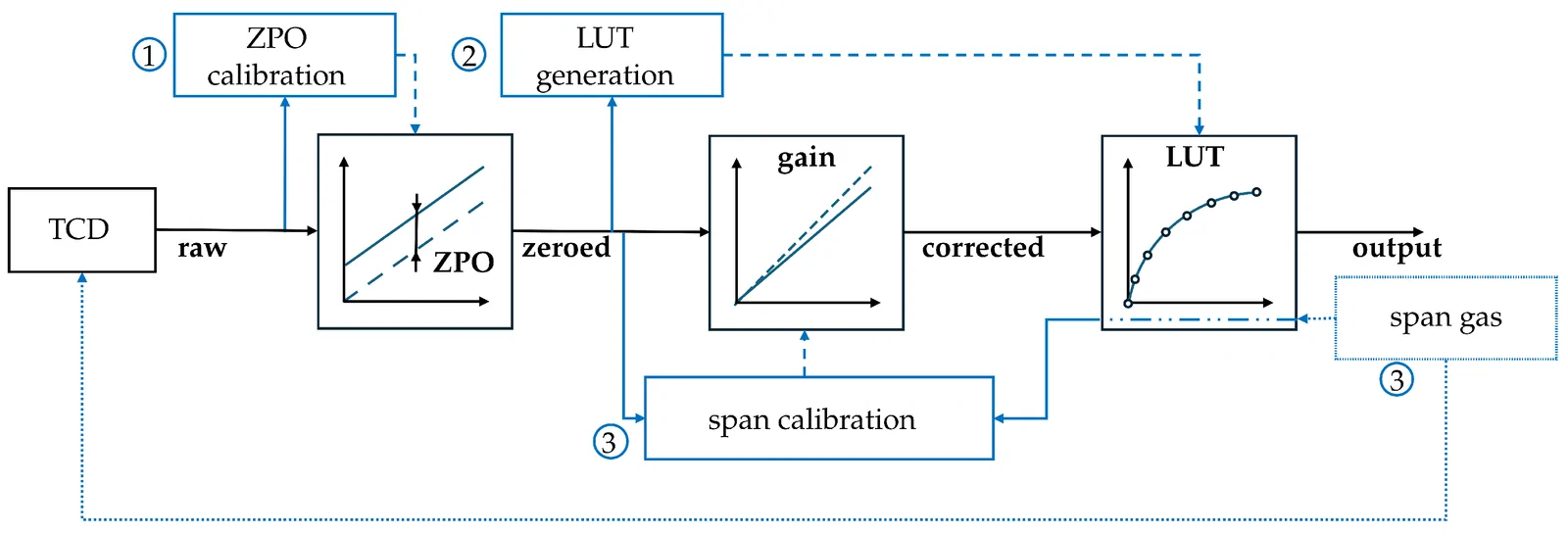
Calibration and data processing scheme for the TCD raw signal. (1) Zero-point offset calibration: the TCD is exposed to the zero gas and the raw value is recorded and stored as the ZPO, which is subsequently subtracted from all raw readings; (2) Look-up-table calibration: using MFCs, gas mixtures of defined concentrations are premixed and their zeroed values are mapped to the corresponding entries in the look-up table; (3) Span calibration: at defined intervals, a gas of known output value is passed through the TCA, from which, a gain factor is calculated and subsequently multiplied by all zeroed readings.

The gain value should always remain close to unity, as it is intended to correct only minor sensitivity deviations. If it deviates too far from this target value, the measurement setup should be inspected and recalibrated if necessary. The complete calibration procedure, along with the steps involved in converting raw values to output values, is shown schematically in Figure 3.

To assess the stability of the measurement signal, a total of 36 measurement cycles are performed over six days using 11 discrete H_2_/N_2_-mixtures. 100 vol.% N_2_ is used as the zero gas while 100 vol.% H_2_ is used as the span gas, corresponding to an expected output of 100%. A stabilization time of 90 s was applied. On each day, six measurement cycles are conducted in which the H_2_ concentration is increased stepwise from 0 vol.% to 100 vol.% in increments of 10 vol.%, with each concentration step held for 90 s, while the thermal conductivity detector raw value is recorded. Upon completion of each cycle, the concentration is reduced to 0 vol.% and a new cycle is initiated. The gas mixtures are produced by combining pure H_2_ and N_2_ using the mass flow controllers at a total volumetric flow rate of ${\dot{V}}_{\mathrm{tot}}=4\frac{l}{\min}$. To evaluate each static measurement point, the raw values are averaged over a 10 s window, positioned such that it ends 3 s prior to the subsequent MFC concentration adjustment.

Three different calibration strategies are investigated:

- **Case (a):** ZPO calibration, LUT generation and span calibration (gain = 1) with data from the first cycle of day one, no recalibration thereafter;
- **Case (b):** LUT generation and span calibration (gain = 1) with data from the first cycle of day one, daily ZPO recalibration with data from the first cycle of every day;
- **Case (c):** LUT generation with data from the first cycle of day one, daily ZPO calibration and span recalibration with data from the first cycle of every day.

The influence of MFC repeatability on the measured hydrogen concentration $x_{H_{2}}$ is determined using Gaussian error propagation. Since the measurement system is calibrated using the same mass flow controllers as those employed in the experiments, systematic offsets cancel and the uncertainty is dominated by the repeatability of the devices. Based on the specifications given in Table 1, the molar fraction and its associated statistical uncertainty $u_{x_{H_{2}}}$ are calculated as:

(2) $$x_{H_{2}}\left({\dot{V}}_{H_{2}},{\dot{V}}_{N_{2}}\right)=\frac{{\dot{V}}_{H_{2}}}{{\dot{V}}_{H_{2}}+{\dot{V}}_{N_{2}}}$$

(3) $$u_{x_{H_{2}}}\left({\dot{V}}_{H_{2}},{\dot{V}}_{N_{2}}\right)=\sqrt{{\left(\frac{\partial x_{H_{2}}}{\partial {\dot{V}}_{H_{2}}}\;\cdot \;u_{{\dot{V}}_{H_{2}}}\left({\dot{V}}_{H_{2}}\right)\right)}^{2}+{\left(\frac{\partial x_{H_{2}}}{\partial {\dot{V}}_{N_{2}}}\;\cdot \;u_{{\dot{V}}_{N_{2}}}\left({\dot{V}}_{N_{2}}\right)\right)}^{2}}$$

The sensitivity coefficients are derived as:

(4) $$\frac{\partial x_{H_{2}}}{\partial {\dot{V}}_{H_{2}}}=\frac{{\dot{V}}_{N_{2}}}{{\left({\dot{V}}_{H_{2}}+{\dot{V}}_{N_{2}}\right)}^{2}}=\frac{1-x_{H_{2}}}{{\dot{V}}_{\mathrm{tot}}}\;\mathrm{and}\;\frac{\partial x_{H_{2}}}{\partial {\dot{V}}_{N_{2}}}=-\frac{{\dot{V}}_{H_{2}}}{{\left({\dot{V}}_{H_{2}}+{\dot{V}}_{N_{2}}\right)}^{2}}=-\frac{x_{H_{2}}}{{\dot{V}}_{\mathrm{tot}}}$$

where $u_{{\dot{V}}_{i}}\left({\dot{V}}_{i}\right)$ denotes the standard uncertainty associated with the repeatability of each MFC. The combined uncertainty can then be expressed as:

(5) $$u_{x_{H_{2}}}\left(x_{H_{2}}\right)=\left\{\begin{array}{cc} 0 & \mathrm{if}\;x_{H_{2}}=0.0\;\mathrm{or}\;x_{H_{2}}=1.0\\ \frac{1-x_{H_{2}}}{2500}\;\cdot \;\sqrt{25x_{H_{2}}^{2}+1} & \mathrm{if}\;0.0<x_{H_{2}}<0.2\\ \frac{2\sqrt{2}}{1000}\;\cdot \;x_{H_{2}}\;\cdot \;\left(1-x_{H_{2}}\right) & \mathrm{if}\;0.2\leq x_{H_{2}}\leq 0.8\\ \frac{x_{H_{2}}}{2500}\;\cdot \;\sqrt{25{\left(x_{H_{2}}-1\right)}^{2}+1} & \mathrm{if}\;0.8<x_{H_{2}}<1.0 \end{array}\right.$$

with ${\dot{V}}_{\mathrm{tot}}$ held constant at full scale of the MFCs, corresponding to ${\dot{V}}_{\mathrm{tot}}=4\frac{l}{\min}$ in the present work.

Subsequently, measurements are performed using a quasi-binary surrogate of the ammonia cracking product gas to verify whether the analyzer performance characterized from the binary H_2_/N_2_ measurements, in terms of repeatability and stability, is transferable to the conversion ratio of an NH_3_ cracker. The conversion ratio $X_{{\mathrm{NH}}_{3}}$ describes the fraction of NH_3_ that has been decomposed into H_2_ and N_2_.

To generate the surrogate mixture, two MFCs are used: one feeds pure ammonia, while the other feeds a premixed cylinder gas representing the product of complete ammonia decomposition (75 vol.% H_2_ and 25 vol.% N_2_). Since the total volumetric flow rate varies with conversion ratio, this effect is accounted for by scaling the total flow from ${\dot{V}}_{\mathrm{tot},\;\mathrm{start}}=2\frac{l}{\min}$ at 0 conversion (100 vol.% NH_3_) to ${\dot{V}}_{\mathrm{tot},\;\mathrm{end}}=4\frac{l}{\min}$ at 100% conversion. The pure ammonia is used as the zero gas and the premixed product gas is used as the span gas, with a desired output of 100% conversion. A stabilization time of 90 s was applied.

Based on the stoichiometry of the ammonia decomposition (2NH_3_ → 3H_2_ + N_2_), the conversion ratio $X_{{\mathrm{NH}}_{3}}$ can be expressed as a function of the measured hydrogen molar fraction $x_{H_{2}}$:

(6) $$X_{{\mathrm{NH}}_{3}}\left(x_{H_{2}}\right)=\frac{x_{H_{2}}}{1.5-x_{H_{2}}}$$

The flow-rate-dependent hydrogen molar fraction

(7) $$x_{H_{2}}\left({\dot{V}}_{H_{2}/N_{2}},{\dot{V}}_{{\mathrm{NH}}_{3}}\right)=0.75\;\cdot \;\frac{{\dot{V}}_{H_{2}/N_{2}}}{{\dot{V}}_{H_{2}/N_{2}}+{\dot{V}}_{{\mathrm{NH}}_{3}}}$$

is used to derive the flow-rate-dependent conversion ratio

(8) $$X_{{\mathrm{NH}}_{3}}\left({\dot{V}}_{H_{2}/N_{2}},{\dot{V}}_{{\mathrm{NH}}_{3}}\right)=\frac{{\dot{V}}_{H_{2}/N_{2}}}{{\dot{V}}_{H_{2}/N_{2}}+2{\dot{V}}_{{\mathrm{NH}}_{3}}}.$$

The associated uncertainty $u_{X_{{\mathrm{NH}}_{3}}}$ is obtained via Gaussian error propagation:

(9) $$\begin{array}{cc} u_{X_{{\mathrm{NH}}_{3}}}\left({\dot{V}}_{H_{2}/N_{2}},{\dot{V}}_{{\mathrm{NH}}_{3}}\right)= & \frac{2}{{\left({\dot{V}}_{H_{2}/N_{2}}+2{\dot{V}}_{{\mathrm{NH}}_{3}}\right)}^{2}}\\ \;\cdot \; & \sqrt{{\left({\dot{V}}_{H_{2}/N_{2}}\;\cdot \;u_{{\dot{V}}_{{\mathrm{NH}}_{3}}}\left({\dot{V}}_{{\mathrm{NH}}_{3}}\right)\right)}^{2}+{\left({\dot{V}}_{{\mathrm{NH}}_{3}}\;\cdot \;u_{{\dot{V}}_{H_{2}/N_{2}}}\left({\dot{V}}_{H_{2}/N_{2}}\right)\right)}^{2}} \end{array}$$

The volumetric flow rates can be expressed as functions of $X_{{\mathrm{NH}}_{3}}$ and the initial volumetric flow rate ${\dot{V}}_{\mathrm{tot},\;\mathrm{start}}$ at 0 conversion as follows:

(10) $${\dot{V}}_{H_{2}/N_{2}}\left(X_{{\mathrm{NH}}_{3}}\right)=2\;\cdot \;X_{{\mathrm{NH}}_{3}}\;\cdot \;{\dot{V}}_{\mathrm{tot},\mathrm{start}}\;\mathrm{and}\;{\dot{V}}_{{\mathrm{NH}}_{3}}\left(X_{{\mathrm{NH}}_{3}}\right)=\left(1-X_{{\mathrm{NH}}_{3}}\right)\;\cdot \;{\dot{V}}_{\mathrm{tot},\mathrm{start}}$$

With ${\dot{V}}_{\mathrm{tot},\;\mathrm{start}}=2\frac{l}{\min}$ and MFC full scale flow rates of ${\dot{V}}_{\mathrm{MFC}\;\max,\;H_{2}/N_{2}}=4\frac{l}{\min}$ and ${\dot{V}}_{\mathrm{MFC}\;\max,\;{\mathrm{NH}}_{3}}=8\frac{l}{\min}$, the conversion ratio uncertainty expressed solely as a function of $X_{{\mathrm{NH}}_{3}}$ itself is:

(11) $$\begin{array}{c} u_{X_{{\mathrm{NH}}_{3}}}\left(X_{{\mathrm{NH}}_{3}}\right)=\left\{\begin{array}{cc} 0 & \mathrm{if}\;X_{{\mathrm{NH}}_{3}}=0.0\;\mathrm{or}\;X_{{\mathrm{NH}}_{3}}=1.0\\ \frac{1-X_{{\mathrm{NH}}_{3}}}{2500}\;\cdot \;\sqrt{25X_{{\mathrm{NH}}_{3}}^{2}+1} & \mathrm{if}\;0.0<X_{{\mathrm{NH}}_{3}}\leq 0.2\\ \frac{X_{{\mathrm{NH}}_{3}}}{2500}\;\cdot \;\sqrt{25X_{{\mathrm{NH}}_{3}}^{2}-50X_{{\mathrm{NH}}_{3}}+41} & \mathrm{if}\;0.2<X_{{\mathrm{NH}}_{3}}<1.0 \end{array}\right. \end{array}$$

As in the preceding analysis, the individual uncertainties $u_{{\dot{V}}_{i}}\left({\dot{V}}_{i}\right)$ are based on the repeatability specifications in Table 1, while systematic offsets are neglected on account of the inherent calibration of the measurement chain.

**Figure 4:**
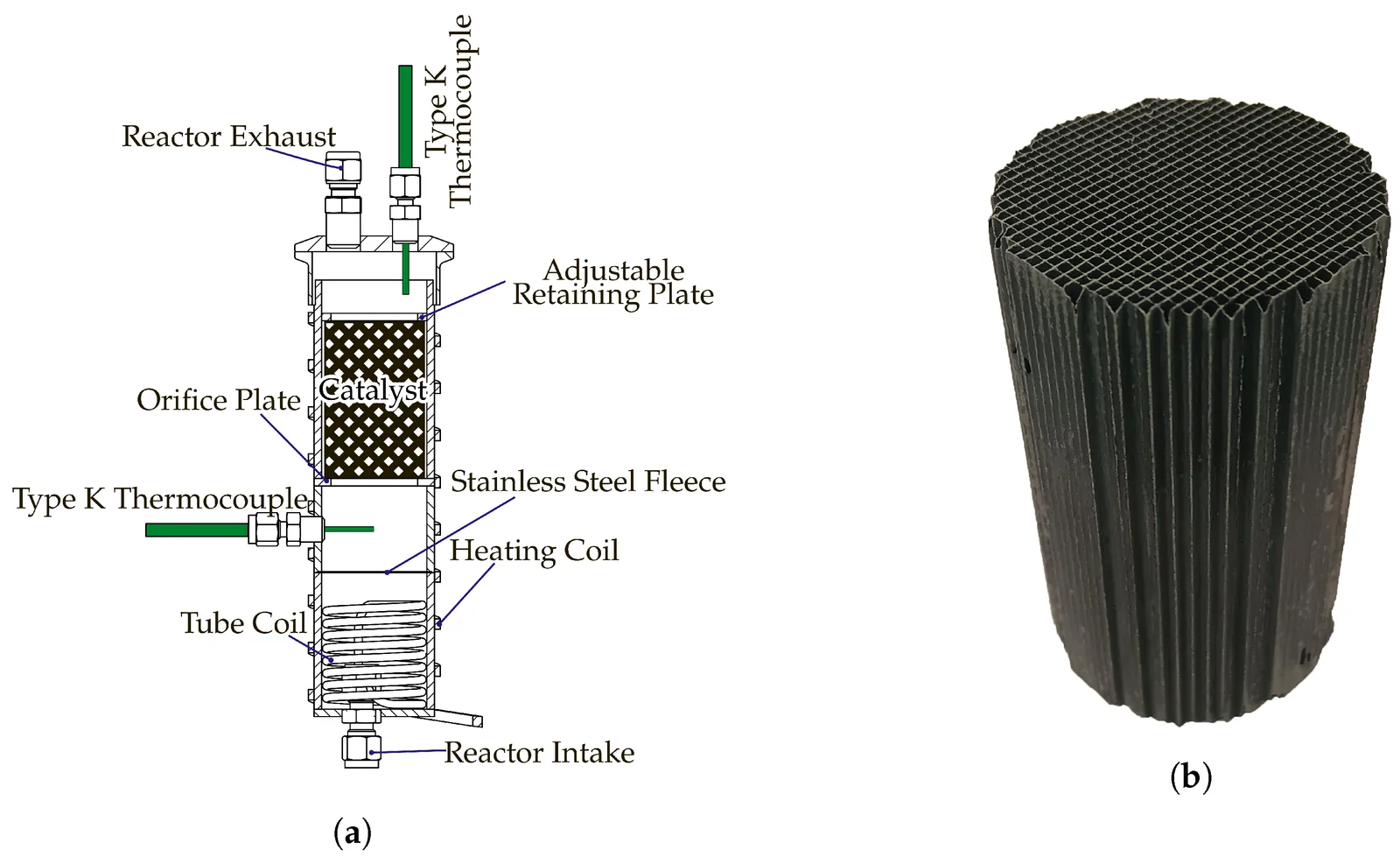
Additional components for catalyst characterization. (**a**) Schematic of the reactor housing. The reactor is heated externally by a heating coil wound around its outer walls. The process gas enters through a tube coil for thermal equilibration, then passes through a stainless steel mesh for flow laminarization before reaching the catalyst, which is positioned downstream of an orifice plate serving as a retainer and enclosed within the reactor housing. A downstream cooling coil not shown here reduces the gas temperature to a level compatible with the TCA. (**b**) Photograph of the monolithic catalyst used in this study. The honeycomb structure provides a high specific geometric surface area while maintaining a low pressure drop across the catalyst bed.

In the final experimental configuration, a temperature-controlled reactor is installed between the MFCs and the TCA, as shown in Figure 1. This reactor is illustrated in Figure 4a. The reactor housing is heated externally via a heating coil, preventing direct contact between the process gas and the coil. Prior to reaching the catalyst, the gas passes through an elongated tube coil, which extends its residence time in the heated zone and ensures thermal equilibration to the target temperature. Upon entering the reactor, the gas first traverses a stainless steel mesh for flow laminarization, followed by an orifice plate serving as a catalyst retainer. The catalyst, shown in Figure 4b, is positioned directly downstream of the orifice plate, enclosed within the reactor housing, and retained axially by the retaining plate on the outlet side. Upon exiting the catalyst bed, the gas passes through a cooling coil, which reduces the gas temperature to a level compatible with the TCA.

Temperature-dependent measurements are conducted by incrementally increasing the heating coil setpoint from 200 °C to 650 °C in steps of 50 °C, at a space velocity of $2100\;\frac{1}{h}$. At each temperature level, the system is allowed to stabilize until both the TCD signal and all monitored temperatures reach steady state, after which measurement data are recorded over an interval of 15 s. The procedure is then reversed, reducing the temperature stepwise back to the initial setpoint. The measured conversion ratio of the catalyst is subsequently compared with the conversion at chemical equilibrium, which is calculated using the Cantera Version 3.2.0 [26] chemoinformatics extension in MatLab R2022b [27].

## 3. Results

### 3.1. H_2_/N_2_-Mixture

**Figure 5:**
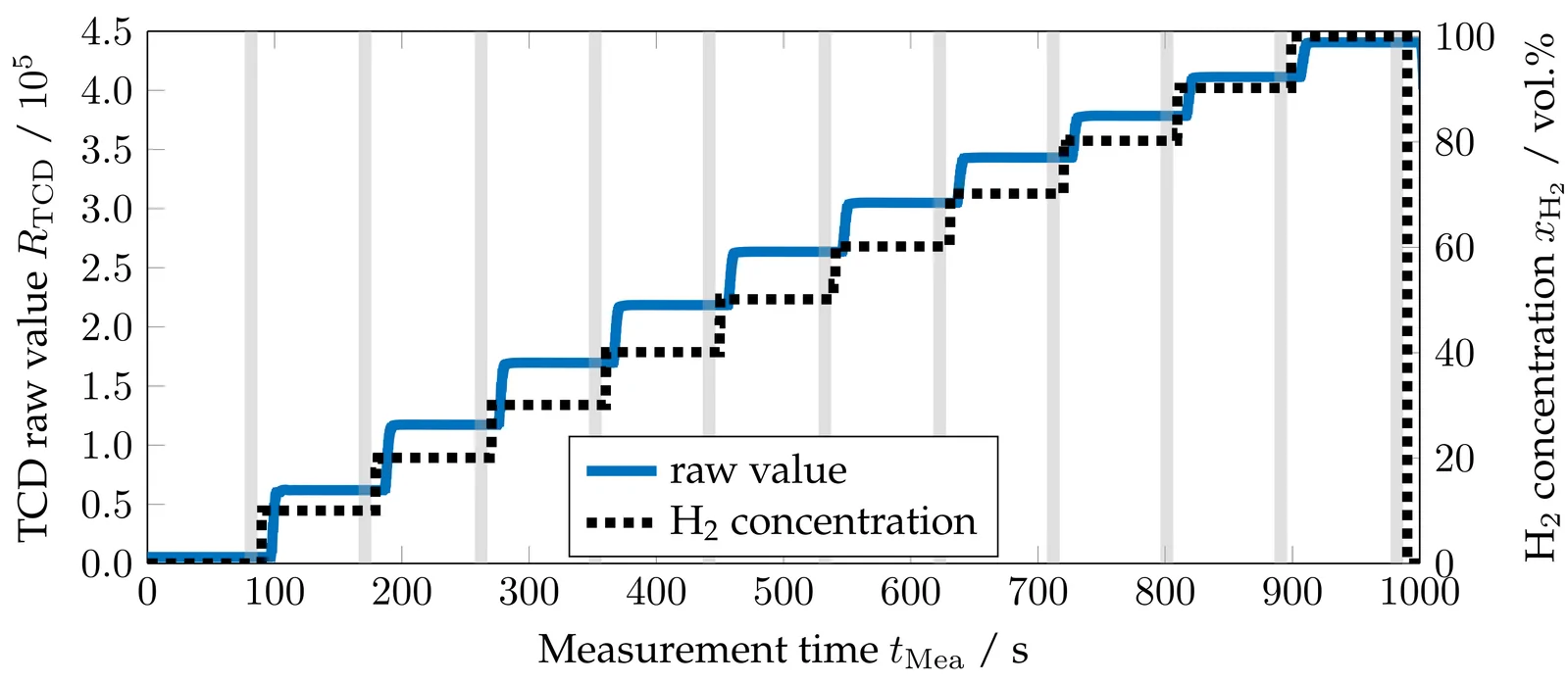
Time evolution of the TCD raw value and hydrogen concentration in the sample gas over the course of a representative measurement cycle. The raw value lags behind the hydrogen concentration by the system response time $t_{90}$ (9.7 s). The gray shaded regions indicate the data windows used for subsequent analysis.

The evolution of the H_2_ concentration and TCD raw value during a representative measurement cycle is shown in Figure 5. The raw value lags behind concentration changes by the system response time $t_{90}$, which averages 9.7 s (range 9.1 s to 10.2 s), indicating a negligible dependence on the H_2_ concentration. It is also apparent that sensitivity decreases with increasing H_2_, as evident by the diminishing step height at higher concentrations. Since the signal noise is several orders of magnitude lower than the raw signal amplitude, the influence of this nonlinear sensitivity reduction on measurement accuracy is negligible, as it is captured by the LUT calibration. The cycle shown is repeated six times on each measurement day. Between cycles, the entire measurement line is purged with N_2_ to prevent measurement errors caused by residual hydrogen.

As described in Section 2, the raw values are averaged over a 10 s window that ends 3 s before the next concentration adjustment. These averaging windows are highlighted in Figure 5. The average raw values are subsequently processed according to the calibration methodology outlined in Figure 3 to obtain the TCA output value. This is carried out separately for each of the three calibration cases introduced earlier, yielding 6 measurement points per concentration level per day and a total of 36 data points per concentration level across all six days.

**Figure 6:**
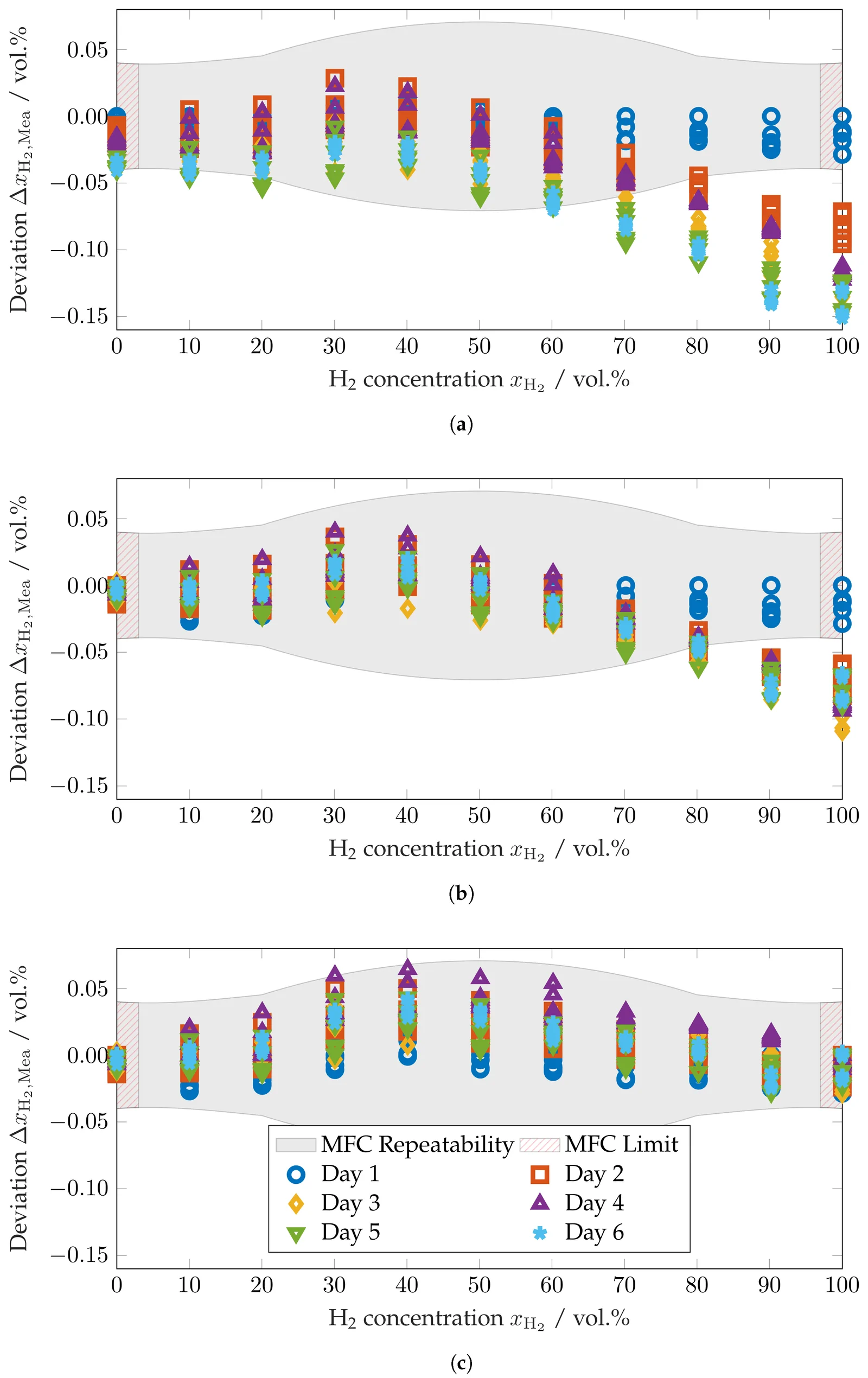
Residuals of the measured hydrogen concentration in vol.% for the three tested calibration strategies. Measurement Cycle 1 from Day 1 is used to generate the LUT in all cases. The legend in (**c**) applies to all panels in this figure. In case (**c**), all residuals fall within the repeatability limits of the MFCs. The red-hatched area indicates the concentration range that cannot be reliably mixed with the MFCs given their flow rate limitations. This does not affect the results at 0 vol.% and 100 vol.%, since only a single MFC is active in those cases, and therefore no mixing-related uncertainty arises. (**a**) No daily recalibration. (**b**) Daily zero-point calibration. (**c**) Daily zero-point and span calibration.

Figure 6 shows the deviations in the TCA output value from the nominal hydrogen concentrations for all 396 datapoints, with a separate panel for each of the three cases. The uncertainty of the mixture concentration generated by the MFCs is indicated in each panel by a gray area. Since the MFCs cannot deliver a reliable flow below 2% of their full-scale capacity, a concentration range exists in which only a theoretical uncertainty can be specified, as these concentrations cannot be physically realized with the current setup. The concentrations of $x_{H_{2}}=0\;\mathrm{vol}.\%$ and $x_{H_{2}}=100\;\mathrm{vol}.\%$ are unaffected by this issue, as only a single MFC is active at each point, eliminating mixing uncertainty.

In Case (a) (Figure 6a), the TCA is fully calibrated on Day 1 with no further corrections applied thereafter. With the exception of two data points, all measurements remain within the mixing uncertainty up to a concentration of $x_{H_{2}}=70\;\mathrm{vol}.\%$. Beyond this threshold, all days except Day 1 exhibit a drift towards lower hydrogen concentrations, reaching a maximum absolute deviation of $\Delta x_{H_{2}}=-0.151\;\mathrm{vol}.\%$ at $x_{H_{2}}=100\;\mathrm{vol}.\%$ on Day 6.

Case (b) (Figure 6b) adds a daily zero-point correction to the initial full calibration. The behavior up to $x_{H_{2}}=70\;\mathrm{vol}.\%$ improves noticeably, with all data points now falling within the mixing uncertainty. The downward drift above this concentration persists on all days except Day 1, though the maximum absolute deviation is reduced to $\Delta x_{H_{2}}=-0.109\;\mathrm{vol}.\%$ at $x_{H_{2}}=100\;\mathrm{vol}.\%$ on Day 3.

In Case (c) (Figure 6c), an additional daily span correction is applied alongside the zero-point correction. This fully eliminates the observed drift, with all data points falling within the mixing uncertainty across the entire concentration range. The maximum absolute deviation is reduced further to $\Delta x_{H_{2}}=+0.064\;\mathrm{vol}.\%$ at $x_{H_{2}}=40\;\mathrm{vol}.\%$ on Day 4.

### 3.2. Synthetic NH_3_ Cracking Gas Mixtures

**Figure A1:**
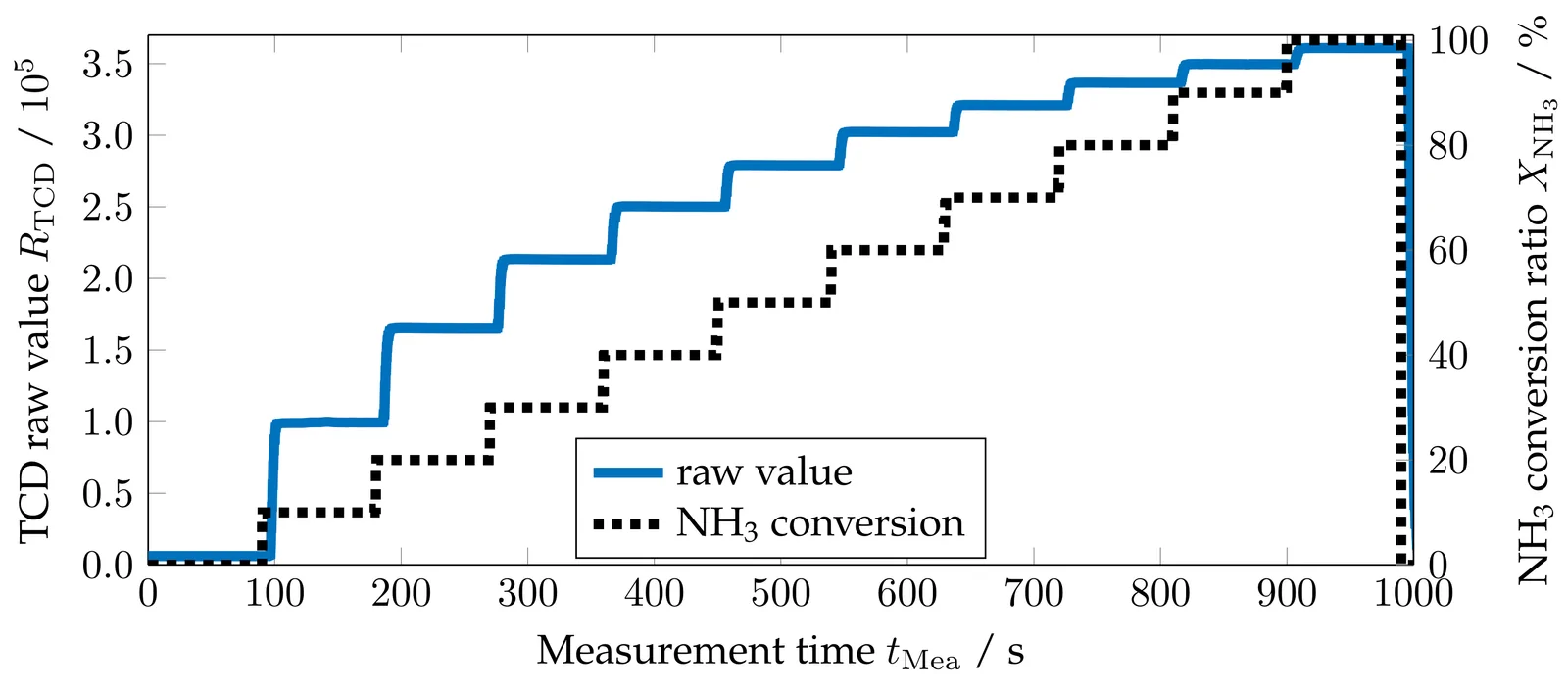
Time evolution of the TCD raw value and the synthetic ammonia conversion ratio over the coures of a representative measurement cycle.

The evolution of the NH_3_ conversion and TCD raw value during a representative measurement cycle is shown in Figure A1. To replicate the gas composition resulting from NH_3_ cracking, the constituent gases are synthetically blended by two MFCs, using pure NH_3_ and a premixed 75 vol.% H_2_/25 vol.% N_2_ cylinder gas. Rather than varying the hydrogen concentration in 10 vol.% increments, the ammonia conversion ratio is varied instead. Owing to the stoichiometry of ammonia cracking, the relationship between these two variables is nonlinear. Table 2 lists the resulting hydrogen concentration for each 10% conversion increment.

As in the previous experiment, six measurement cycles, in which the conversion ratio is increased in 10% increments from 0% to 100%, are again performed on each of six measurement days. The TCD raw value is again averaged over a 10 s window that ends 3 s prior to the next change in gas concentration.

**Figure 7:**
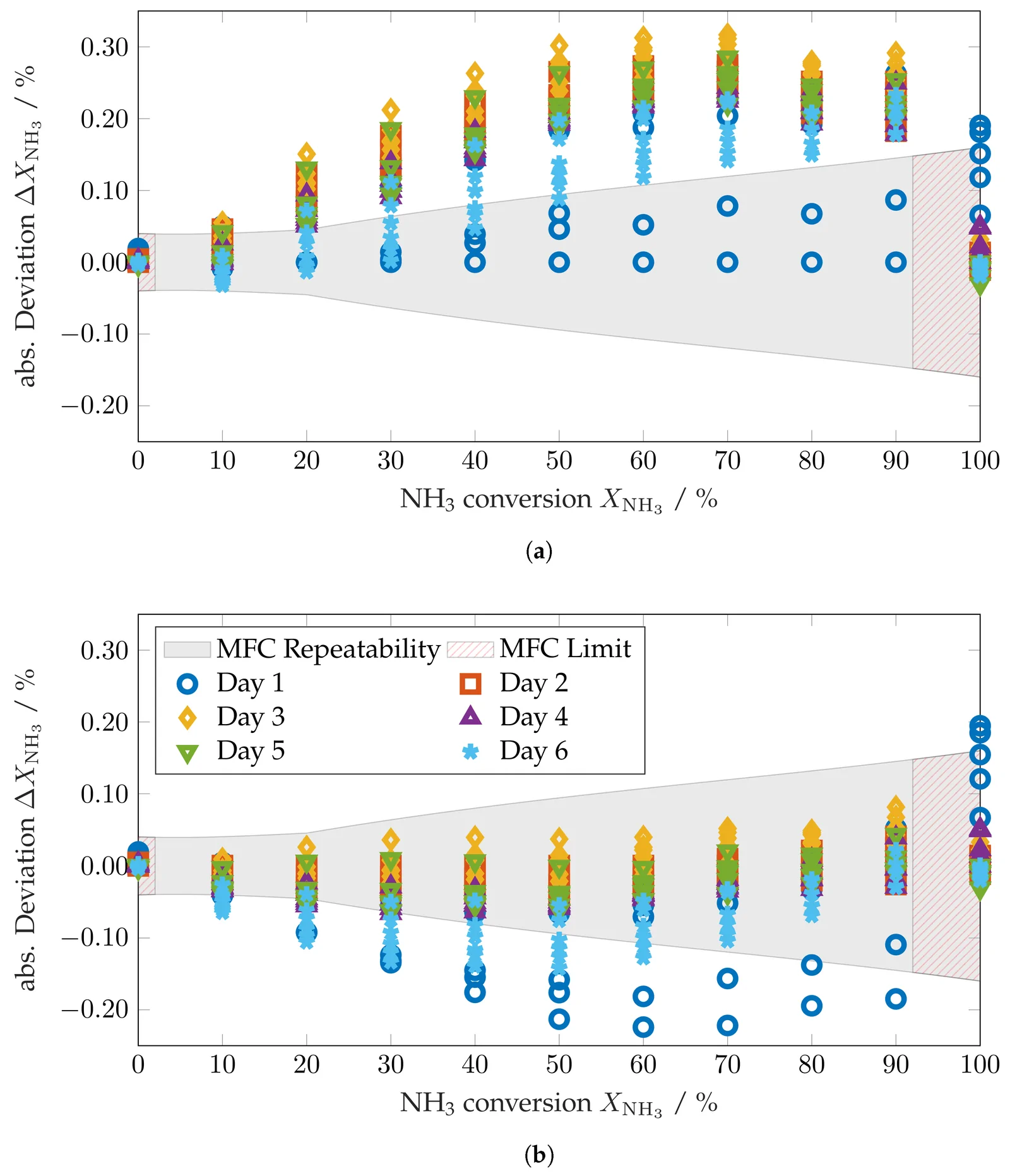
Measurement residuals of the ammonia conversion ratio, in percentage points, classified by LUT generation day. A clear difference in linearity is evident between Day 1 and the remaining days. The red-hatched area indicates the conversion ratio range that cannot be reliably mixed by the MFCs given their flow rate limitations. This does not affect the results at 0% and 100%, since only a single MFC is active in those cases, and therefore no mixing-related uncertainty arises. The legend in (**b**) applies to both panels: (**a**) LUT generated from Day 1. (**b**) LUT generated from Day 2.

Figure 7 shows the deviation of the TCA output value from the nominal conversion ratio set by the MFCs. The calibration method corresponds to the procedure already described for Case (c) in Figure 6c, with zero-point and span calibration performed daily. The region corresponding to the mixing uncertainty caused by the MFCs is shaded gray. Unlike in the diagrams in Figure 6, this region is not symmetric. Instead, the uncertainty increases with increasing conversion ratio. This asymmetry arises from the nonlinear relationship between hydrogen concentration and ammonia conversion ratio, as given by Equation (6), as well as the different full-scale capacities of the MFCs. The two red-shaded areas highlighting the conversion ratios that cannot be reliably mixed by the MFCs are likewise not of equal width, also owing to the differing full-scale capacities of the two devices: the NH_3_-MFC is set to ${\dot{V}}_{\max,{\mathrm{NH}}_{3}}=8\frac{l}{\min}$, while the H_2_/N_2_-MFC is set to ${\dot{V}}_{\max,H_{2}/N_{2}}=4\frac{l}{\min}$. Accordingly, the immiscible region is wider at low ammonia concentrations, i.e., high conversion ratios. The data at 0% and 100% conversion are unaffected by this, since only a single MFC is active at each of these respective points.

Figure 7a shows the measured values when the LUT is generated using measurement Cycle 1 on Day 1. All remaining cycles exhibit a significant upward deviation from this calibration that cannot be explained by the mixture uncertainty alone. With the exception of the second measurement cycle on Day 1, every other cycle on Day 1 is likewise affected by this phenomenon.

In light of this, the entire procedure is repeated with the LUT generated from a different day. Measurement Cycle 1 from Day 2 is selected for this purpose as it lies at sufficient temporal distance from the original calibration to reveal whether the observed deviation is attibutable to a drift in sensor response rather than to a systematic error in the calibration procedure itself. The resulting measured values are shown in Figure 7b. The majority of data points now fall within the uncertainty range, with the exception of the first two measurement cycles from Day 1, as well as a number of data points from measurement Day 6. This improvement supports the assumption that the deviations observed in Figure 7a originate primarily from a drift-related offset in the calibration reference rather than from a fundamental limitation of the measurement principle.

**Figure 8:**
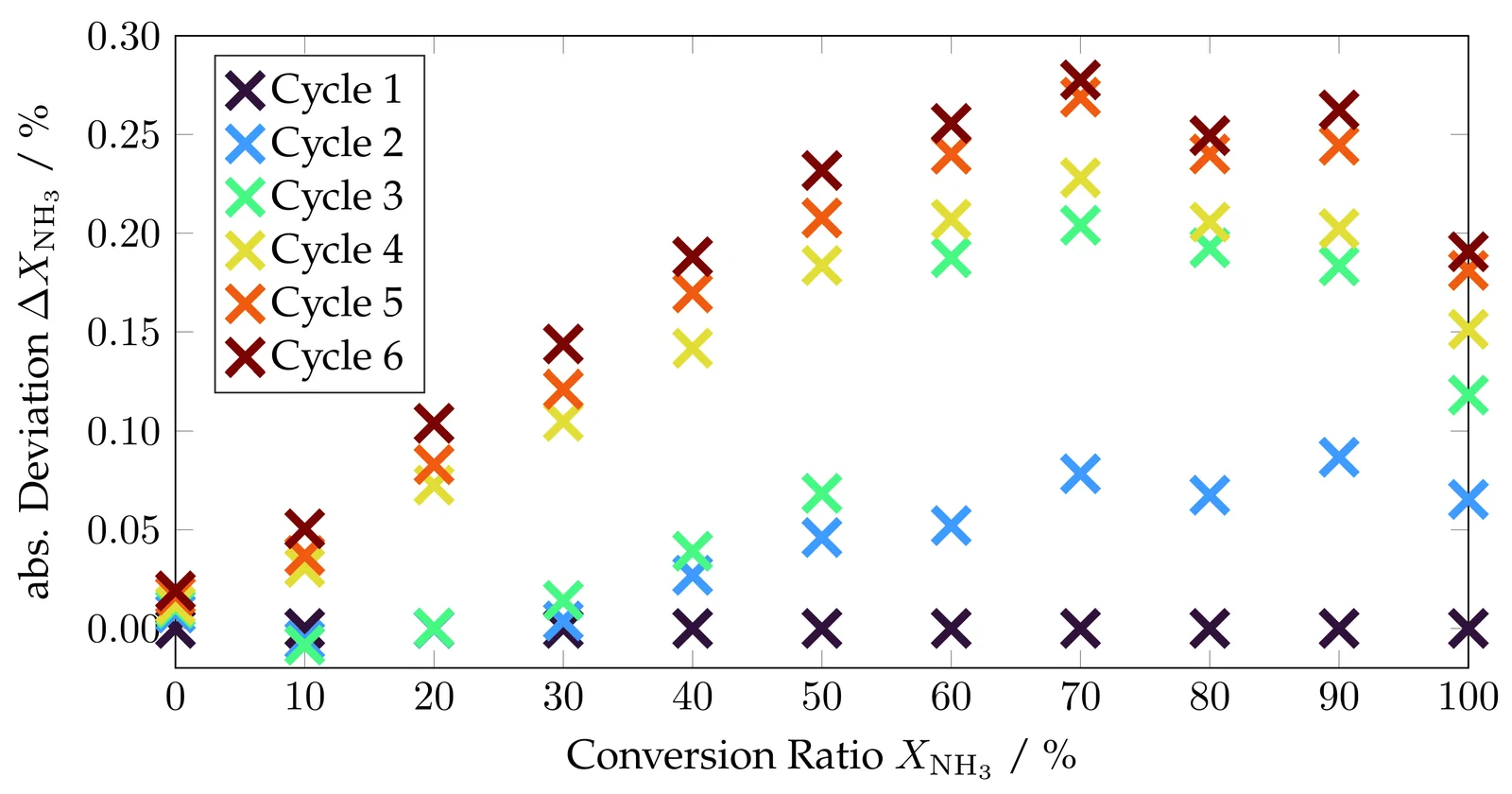
Measurement residuals from the synthetic NH_3_ cracking gas characterization on Day 1, classified by measurement cycle. The LUT is generated using data from Cycle 1. Two distinct segments are observed in the residuals: the first comprises Cycles 1 and 2, together with Cycle 3 up to a conversion ratio of $X_{{\mathrm{NH}}_{3}}=50\%$; the second comprises all subsequent data points and cycles, which exhibit a different nonlinearity.

The anomalies observed in the first two measurement cycles of Day 1 are examined in greater detail. The data can be divided into two distinct segments: the first comprises Cycles 1 and 2, together with the data points of Cycle 3 up to and including 50% conversion; the second comprises the remaining Cycle 3 data points, along with Cycles 4, 5, and 6. The residuals of all measurement cycles from Day 1 are plotted relative to Cycle 1 in Figure 8.

### 3.3. Proof-of-Concept: Application to a Monolithic NH_3_ Cracking Catalyst

**Figure A2:**
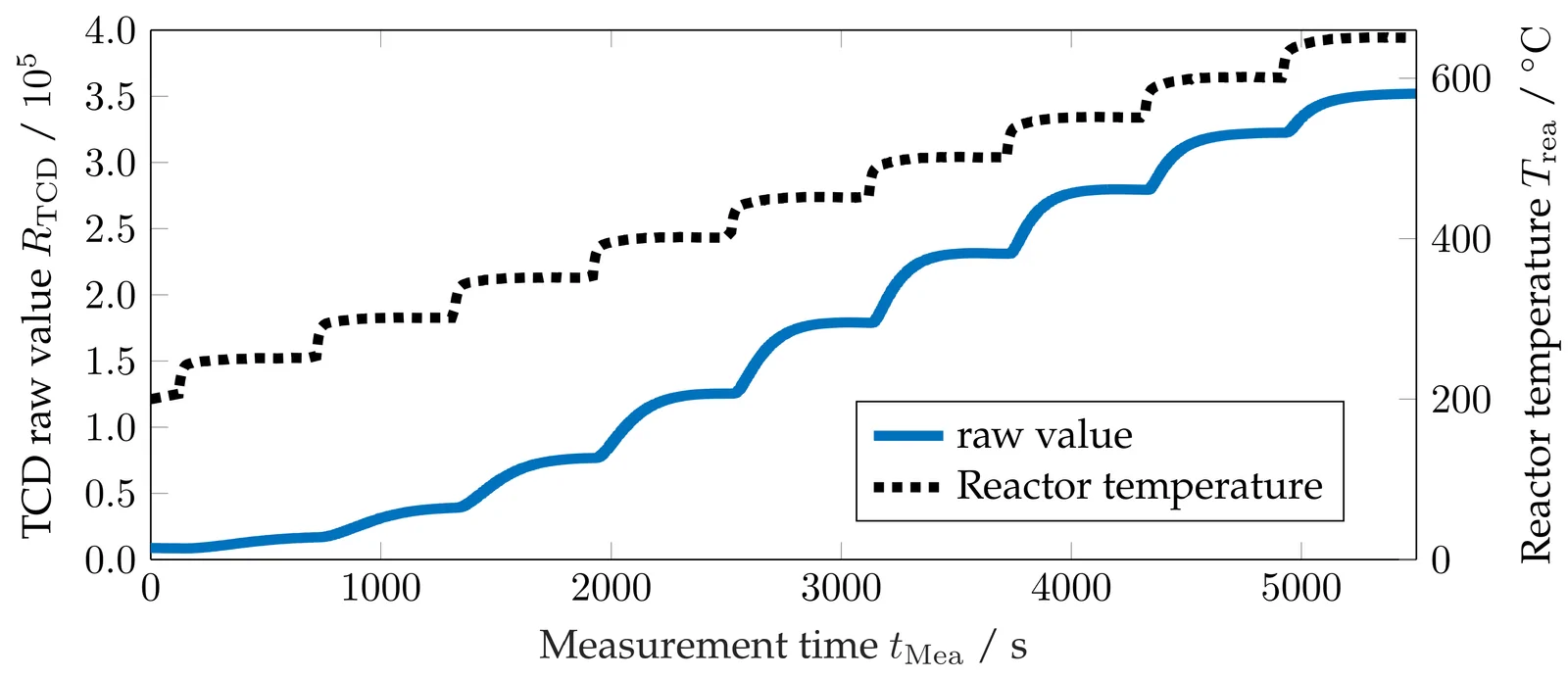
Time evolution of the TCD raw value and the reactor temperature over the course of the one temperature ramp (only half the cycle is shown for better visiblity; the temperature subsequently decreases in equivalent steps).

To demonstrate the applicability of the developed TCA under realistic cracking conditions, a preliminary characterization of a monolithic catalyst was performed. The evolution of the TCD raw value and the reactor temperature during measurement is shown in Figure A2. This experiment is not intended as a comprehensive catalyst study, but rather as a functional validation of the measurement chain in an end-to-end cracking scenario. The LUT used to characterize the cracking catalyst is identical to that used in Figure 7b. Prior to the catalyst measurement, a zero-point calibration and a span calibration are performed. The maximum deviations identified in Figure 7 are used as the confidence interval for this measurement, corresponding to a deviation of +0.317 percentage points upward (Figure 7a) and −0.224 percentage points downward (Figure 7b).

**Figure 9:**
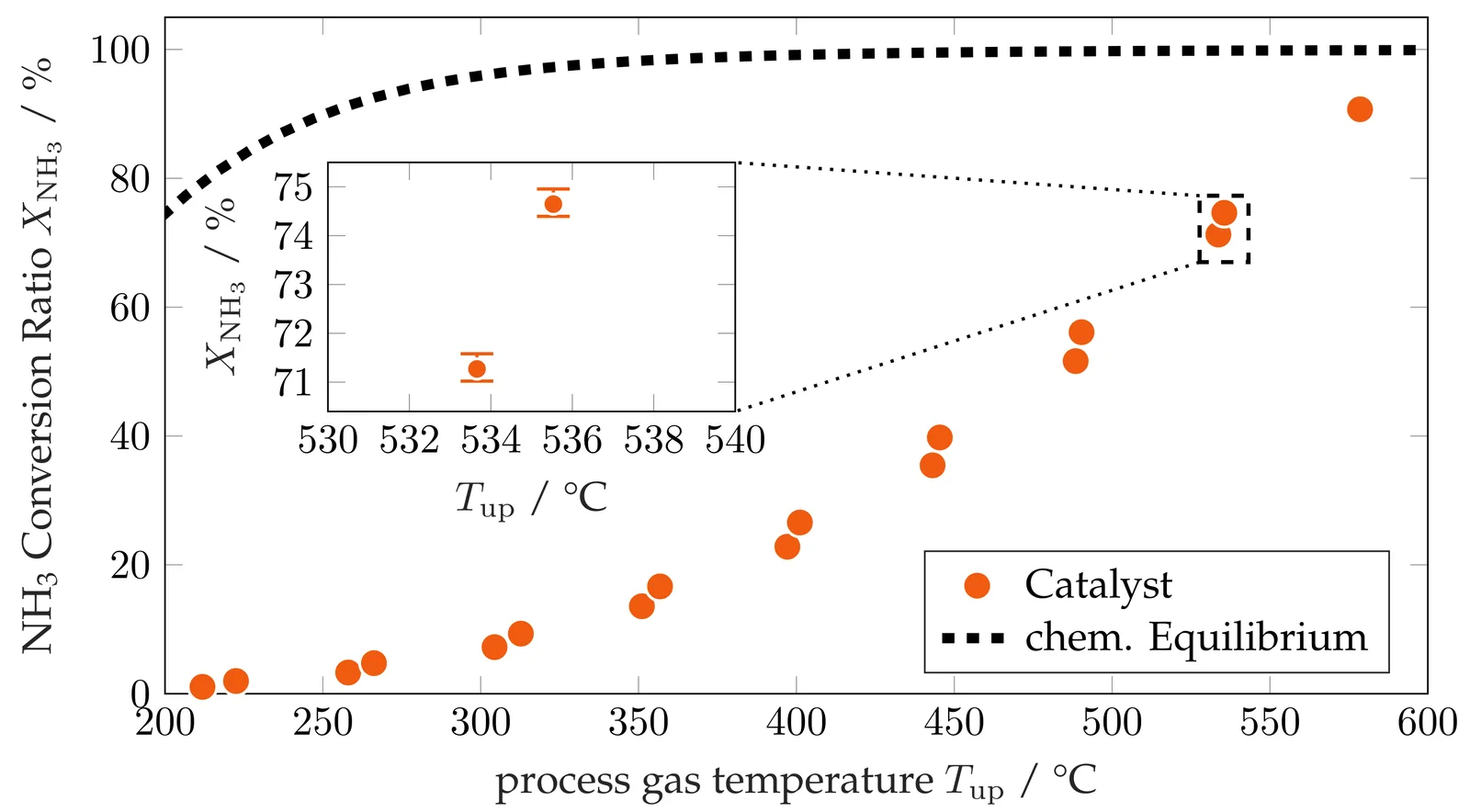
Conversion ratio of the catalyst as a function of process gas temperature. The worst-case deviations from Figure 7 are used as confidence intervals. Since these intervals are too small to be visible on a scale of 0–100%, the figure includes an inset showing a magnified view of the intervals.

Figure 9 shows the conversion ratio of the tested catalyst as a function of reactor temperature. The confidence interval is too small to be discernible at the scale of the ordinate. An inset is therefore included, showing a magnified view of the error bars.

The chemical equilibrium approaches a conversion ratio of 100% at significantly lower temperatures than the catalyst-mediated reaction. Under the experimental conditions, a maximum conversion of $X_{{\mathrm{NH}}_{3},\mathrm{cat}}=90.97\%$ is achieved over the catalyst at $T_{\mathrm{up}}=578.5$ °C. A conversion ratio of 20%, at which, according to Table 2, the product gas already contains 25% H_2_, is exceeded below 400 °C. A slight hysteresis is observable between heating and cooling ramps: the data points recorded during temperature decrease exhibit a marginally higher conversion ratio than those recorded during temperature increase.

## 4. Discussion

The results shown in Figure 6 indicate that the developed analyzer is well suited for measuring hydrogen in nitrogen. Even with a single calibration, as shown in Figure 6a, the measurements yield a maximum deviation of $\Delta x_{H_{2}}=-0.151\;\mathrm{vol}.\%$, which is sufficient for monitoring a process gas stream in a larger-scale plant. With daily zero-point and span calibration, as shown in Figure 6c, the resulting residuals do not allow the measurement uncertainty of the device itself to be clearly determined within the present experimental setup, since the MFCs used for calibration may not be sufficiently accurate to fully resolve the TCA’s own measurement uncertainty in the present experimental setup.

The results of the synthetic NH_3_ cracking gas measurements in Figure 7 show greater scatter among individual data points, as well as larger deviations from the band defined by the MFC mixing uncertainty. One possible explanation is the combined effect of inter-gas interactions and a decrease in sensitivity at high conversion ratios: N_2_ and NH_3_ possess comparatively similar thermal conductivities (31.6 $\frac{\mathrm{mW}}{\mathrm{mK}}$ for NH_3_ and 30.1 $\frac{\mathrm{mW}}{\mathrm{mK}}$ for N_2_ at a pressure of 1 bar and a detector temperature of 86 °C [24]) and the slope of the hydrogen concentration curve decreases with increasing conversion ratio, as shown in Table 2. Nevertheless, the resulting measurement uncertainties remain relatively small. It is noteworthy that, as with H_2_ in N_2_, the Day 1 measurements of the synthetic NH_3_ cracking gas characterization, particularly the early measurements of that day, deviate significantly from the behavior of the remaining measurements as illustrated in Figure 8. For H_2_ in N_2_, this effect is compensated by the daily calibration. However, no equivalent compensation is available for the synthetic NH_3_ cracking gas measurements.

Two possible explanations are proposed. First, hydrogen may accumulate somewhere along the measurement path during the initial measurements, with the detector reading the correct value only once the local hydrogen concentration has reached saturation. Second, a prolonged measurement pause may allow certain surfaces within the device to oxidize. These oxide layers are then reduced by hydrogen during the first subsequent measurement. McMinn [28] reports that hydrogen reduces the oxide coating on the TCD filament, thereby altering the filament response and, consequently, the TCD raw signal. It is therefore recommended that the device be conditioned by operating it with the measurement gases for an extended period before commencing measurements.

Despite these promising results, several limitations should be acknowledged. The system is calibrated and validated using the same mass flow controllers, so the reported uncertainties reflect precision rather than absolute accuracy. An independent reference analyzer would be required to fully characterize systematic uncertainties. Additionally, long-term stability beyond the six-day assessment period has yet to be characterized.

The catalyst experiment primarily serves to validate the system’s suitability for end-to-end deployment. A rigorous catalyst comparison would require standardized conditions across multiple samples, which is precisely the use case this system is designed to enable in future work. Nevertheless, it should be noted that the catalyst tested here exhibits a temperature-conversion-ratio characteristic curve comparable to other catalysts tested for NH_3_ cracking, such as lithium imides [18] or various nickel- and ruthenium-based catalysts [29]. To conduct a reliable comparison of catalysts, additional samples would need to be installed in the reactor and measured using the analyzer presented here. The TCA is well suited to such measurements, as it is highly resistant to the corrosive and aggressive effects of the process gases and, with regular calibration, enables precise measurement of conversion ratio or H_2_ content.

## 5. Conclusions

An ammonia-resistant Thermal Conductivity Analyzer was developed and validated for in situ monitoring of ammonia cracking processes. Combining an OEM TCD with a chemically resistant bypass line and a three-stage calibration procedure (ZPO, LUT, span), the system measured H_2_ concentrations reliably across 0 vol.% to 100 vol.%. For binary H_2_/N_2_ mixtures, daily zero-point and span recalibration reduced residuals to within the repeatability limits of the calibration MFCs. Measurements on the quasi-binary NH_3_ cracking surrogate showed larger scatter, attributed to inter-gas interactions. An early-cycle anomaly, likely caused by H_2_ accumulation or oxide-layer reduction on the TCD filament, is mitigated through an extended gas-conditioning period. A proof-of-concept characterization of a monolithic NH_3_ cracking catalyst at 200 °C to 650 °C confirmed the TCA’s suitability for end-to-end operation, achieving conversion ratio deviations of +0.317/−0.224 percentage points and a temperature-dependent behavior consistent with literature catalysts. These results demonstrate that thermal conductivity measurement, using suitably resistant materials, offers a simple and robust alternative for continuous ammonia cracking monitoring. While TCD-based sensing of ammonia has been explored in other contexts, such as trace-level detection in the ppm range [30], the present work addresses the distinct challenge of continuous, quantitative process gas monitoring during ammonia cracking at industrially relevant, vol.%-scale concentration ranges. Future work should include validation against an independent reference method, assessment of long-term stability beyond the six-day test period, and application of the TCA to a systematic comparison of different cracking catalysts. Additionally, the root cause of the Day 1 anomaly should be clarified. 

## Figures and Tables

**Table 1:** Repeatability specifications for the MFCs.

Range	Uncertainty $u_{{\dot{V}}_{i}}\left({\dot{V}}_{i}\right)$
0% to 20% of full scale	<±0.04% of full scale
20% to 100% of full scale	<±0.20% of reading

**Table 2:** Hydrogen, nitrogen and ammonia concentrations corresponding to the ammonia conversion ratio in 10% increments.

NH_3_ Conversion Ratio	H_2_ Concentration	N_2_ Concentration	NH_3_ Concentration
$X_{{\mathrm{NH}}_{3}}$/%	$x_{H_{2}}$/vol.%	$x_{N_{2}}$/vol.%	$x_{{\mathrm{NH}}_{3}}$/vol.%
0	0.00	0.00	100.00
10	13.64	4.55	81.82
20	25.00	8.33	66.67
30	34.62	11.54	53.85
40	42.86	14.29	42.86
50	50.00	16.67	33.33
60	56.25	18.75	25.00
70	61.76	20.59	17.65
80	66.67	22.22	11.11
90	71.05	23.68	5.26
100	75.00	25.00	0.00

## Data Availability

The original contributions presented in this study are included in the article. Further inquiries can be directed to the corresponding author.

## Abbreviations

The following abbreviations are used in this manuscript:

ILUT	inverse look-up table
LUT	look-up table
MFC	Mass Flow Controller
TCA	Thermal Conductivity Analyzer
TCD	Thermal Conductivity Detector
ZPO	zero-point offset
